# Inverting CEST or $R_{1\rho }$ data to generate solute spectra without a priori assumptions

**DOI:** 10.1002/mrm.70111

**Published:** 2025-10-14

**Authors:** Daniel F. Gochberg

**Affiliations:** ^1^ Vanderbilt University Institute of Imaging Science Nashville Tennessee USA; ^2^ Department of Radiology and Radiological Sciences Vanderbilt University Medical Center Nashville Tennessee USA; ^3^ Deparment of Physics and Astronomy Vanderbilt University Nashville Tennessee USA

**Keywords:** CEST, molecular imaging, $R_{1\rho }$

## Abstract

**Purpose:**

The goal of CEST imaging is to reveal the underlying tissue composition and exchange. However, quantitative CEST analyses typically require strong assumptions about this same composition and exchange. The goal of this paper is to introduce a new CEST analysis (also applicable to rotating frame relaxation rate $R_{1\rho }$ studies) that reveals the underlying spectrum of exchanging solutes without assuming their number, offsets, or exchange rates.

**Theory and Methods:**

Each exchanging solute adds an additional term to $R_{1\rho }$, and this linearity is leveraged by applying established methods for solving linear problems when the solution is non‐negative and sparse. This signal inversion is demonstrated in simulations and experiments on phantoms.

**Results:**

The simulation and experimental results demonstrate the ability to qualitatively characterize the number of exchanging solutes, their offsets, and their exchange rates, and to do so under difficult fitting conditions such as when multiple solutes are at the same frequency offset. This signal inversion is only grossly accurate and has significant biases, limited range of applicability, and spurious results.

**Conclusion:**

An inversion of the CEST signal is demonstrated that requires minimal assumptions and can qualitatively characterize the distribution of contributing solutes. These results demonstrate proof of concept, but further work is needed to address in vivo conditions, optimize acquisition and analysis methods, and determine trade‐offs between sensitivity, accuracy, range of applicability, and prevalence of fitting artifacts. The ultimate ability of the method to characterize the full range of exchanging solutes in vivo is not yet clear.

## 1 INTRODUCTION

CEST is an imaging method based on the exchange of magnetization between one or more solutes and water, and it is the corresponding tissue characterization that makes CEST valuable. CEST has found in vivo application imaging many different endogenous metabolites (e.g., glutamate,^1^ glycogen,^2^ glycosaminoglycan,^3^ myo‐inositol,^4^ creatine,^5^ phosphocreatine,^6^, ^7^ γ‐aminobutyric acid,^8^, ^9^ urea,^10^, ^11^ carnosine,^12^ lactate,^13^, ^14^ etc.). Further, the role of many additional potential contributors to the in vivo CEST spectrum is an area of active research.^15^, ^16^, ^17^, ^18^, ^19^ However, CEST peaks are broad and overlapping, making the underlying drivers of the CEST signal unclear and requiring strong tissue modeling assumptions for metabolically specific imaging. For example, despite the large number of known exchanging solutes, each individual analysis typically assumes signal contributions from only a small number of endogenous compounds. Although this seeming inconsistency in tissue modeling can be justified by assuming a priori knowledge of tissue composition and solute exchange and relaxation characteristics, such assumptions may not be justified in vivo. For example, translating the promising in vitro results^2^ detecting glycogen by hydroxyl exchange has been confounded by the many solute contributions to the CEST signal in vivo.^20^ Even when a solute is the single biggest contributor to the CEST signal, strong modeling assumptions may reduce the accuracy of the tissue characterization and may not recognize, for example, when multiple solutes contribute at a single frequency offset. Simply speaking, the contributors to CEST signals are often unknown, yet assumed.

The underlying reason CEST analyses make strong assumptions (like constraining the number of exchanging pools) is the difficulty in inverting the CEST signal. Many different combinations of exchanging solutes can produce similar CEST results and constraining the number of solute pools (and often the offsets too) is necessary for non‐linear least square parameter fittings or interpreting commonly applied calculated metrics, such as those based on asymmetry.

The current work presents a new analysis that characterizes tissue composition without requiring strong assumptions. It leverages the linearity of the contributions of exchanging solutes to the relaxation rate in the rotating frame, $R_{1\rho }$, and uses methods for inverting such linear problems, even when underdetermined, by assuming that the spectrum of solute concentrations is non‐negative and often sparse. Unlike conventional CEST analyses, no a priori restrictions on the number, exchange rates, or offsets is applied (although choices in experiment and analysis design may still affect the fitted pool distribution). Below we demonstrate proof of concept for this new approach using simulations and experiments on phantoms. (Although the article focuses on analyzing CEST data, because it is more typically acquired at a range of frequency offsets, analyzed in terms of specific solutes, and is less susceptible to experimental artifacts,^21^, ^22^ the new analysis is also applicable to $R_{1\rho }$ studies, and one such simulation is included).

## 2 THEORY

A CEST experiment measures the water z‐magnetization as a function of the irradiation frequency offset, called a Z‐spectrum. Oftentimes, Z‐spectra are acquired under multiple irradiation powers. After a sufficiently long irradiation time, the measured signal is^23^, ^24^, ^25^:

(1) $$Z_{a}^{\mathrm{ss}}=\frac{\cos^{2}\theta R_{1}}{R_{1\rho }},$$

where the “a” subscript refers to water with normalized steady‐state z‐magnetization $Z_{a}^{\mathrm{ss}}$ and measured longitudinal relaxation rate $R_{1}$. $\cos^{2}\theta =\frac{∆\omega^{2}}{\omega_{1}^{2}+∆\omega^{2}}$ with irradiation frequency offset (from the water resonance) ∆ω and amplitude $\omega_{1}$, both in radians per second. This relation can be inverted to give $R_{1\rho }$ as a function of $Z_{a}^{\mathrm{ss}}$:

(2) $$R_{1\rho }=\frac{\cos^{2}\theta R_{1}}{Z_{a}^{\mathrm{ss}}}.$$

The key relation that facilitates the proposed method is the linear relationship between $R_{1\rho }$ and the individual solute contributions:

(3) $$R_{1\rho }=R_{\mathrm{eff}}+R_{\mathrm{ex}}^{b}+R_{\mathrm{ex}}^{c}+\ldots +{R_{\mathrm{ex}}^{\mathrm{mt}}+R}_{\mathrm{ex}}^{\mathrm{NOE}1}+R_{\mathrm{ex}}^{\mathrm{NOE}2}+\ldots ,$$

where $R_{\mathrm{eff}}$ is the contribution from direct saturation and $R_{\mathrm{ex}}^{b}+R_{\mathrm{ex}}^{c}+\ldots +{R_{\mathrm{ex}}^{\mathrm{mt}}+R}_{\mathrm{ex}}^{\mathrm{NOE}1}+R_{\mathrm{ex}}^{\mathrm{NOE}2}+\ldots$ is the contribution from exchange. The direct saturation term is a function of the water pool relaxation rates and the acquisition parameters ∆ω and $\omega_{1}$ and is independent of the exchanging pools. The exchange component is a sum of contributions from exchanging solutes (pools b, c, …) and one or more macromolecules (pools mt, NOE1, …) with large transverse relaxation rates $R_{2}$, including both large “magnetization transfer” (MT) and smaller nuclear Overhauser effects (NOE)^18^ terms. (The equations below include only one MT pool ($R_{\mathrm{ex}}^{\mathrm{mt}}$) for succinctness, but the fitting formalism allows for multiple large $R_{2}$ pools.) The essential point is that all the contributors to $R_{1\rho }$ in Eq. (3) add linearly.

Following the results and notation of Zaiss and Bachert,^23^ the direct effect is

(4) $$R_{\mathrm{eff}}=R_{1}\cos^{2}\theta +R_{2}\sin^{2}\theta .$$

$R_{2}$ is the transverse relaxation rate of water, and $\sin^{2}\theta =\frac{\omega_{1}^{2}}{\omega_{1}^{2}+∆\omega^{2}}$. Assuming small solute $R_{2}$, the contribution from solute pools has the form

(5) $$R_{\mathrm{ex}}^{b}=f_{b}k_{b}\frac{\delta \omega_{b}^{2}}{\omega_{1}^{2}+\Delta \omega^{2}}\frac{\omega_{1}^{2}}{\omega_{1}^{2}+k_{b}^{2}+{\left(\Delta \omega -\delta \omega_{b}\right)}^{2}}.$$

The MT pool, with its large $R_{2}$, will be modeled with the more complete analytic solution

(6) $$\begin{array}{cc} R_{\mathrm{ex}}^{\mathrm{mt}} & =f_{\mathrm{mt}}\left(k_{\mathrm{mt}}\frac{\delta \omega_{\mathrm{mt}}^{2}}{\omega_{1}^{2}+\Delta \omega^{2}}\frac{\omega_{1}^{2}}{\frac{1}{4}\Gamma^{2}+{\left(\Delta \omega -\delta \omega_{\mathrm{mt}}\right)}^{2}}\right.\\ & \;\left.+\;R_{2\mathrm{mt}}\frac{\omega_{1}^{2}}{\frac{1}{4}\Gamma^{2}+{\left(\Delta \omega -\delta \omega_{\mathrm{mt}}\right)}^{2}}+k_{\mathrm{mt}}\sin^{2}\theta \frac{R_{2\mathrm{mt}}\left(R_{2\mathrm{mt}}+k_{\mathrm{mt}}\right)}{\frac{1}{4}\Gamma^{2}+{\left(\Delta \omega -\delta \omega_{\mathrm{mt}}\right)}^{2}}\right), \end{array}$$

where

(7) $$\Gamma =2\sqrt{\frac{R_{2\mathrm{mt}}+k_{\mathrm{mt}}}{k_{\mathrm{mt}}}\omega_{1}^{2}+\left(R_{2\mathrm{mt}}+k_{\mathrm{mt}}\right)}.$$

k, δω, and $R_{2}$ are the exchange rate, resonant frequency separation from the water resonance, and transverse relaxation rate, respectively, of the exchanging pool indicated by the subscript.

Equations ((3), (4), (5), (6), (7)) indicate that $R_{1\rho }$ can be written as a linear weighting of terms, each of which is a function of the acquisition parameters and zero, two, or three sample parameters, corresponding to contributions from direct saturation, CEST solutes, and the MT macromolecular pool, respectively:

(8) $$\begin{array}{cc} R_{1\rho } & =R_{1}\;\underset{\text{func of}\;\mathrm{acq}\;\text{params}}{\underbrace{g_{\mathrm{co}s^{2}}\left(\omega_{1},\Delta \omega \right)}}+R_{2}\;g_{\mathrm{si}n^{2}}\left(\omega_{1},\Delta \omega \right)\\ & \;+f_{b}\;\underset{\text{func of}\;\mathrm{acq}\;\text{and}\;2\;\text{sample params}}{\underbrace{g_{\text{solute}}\left(\omega_{1},\Delta \omega ,k_{b},\delta \omega_{b}\right)}}+\cdots \\ & \;+f_{\mathrm{mt}}\;\underset{\text{func of}\;\mathrm{acq}\;\text{and}\;3\;\text{sample params}}{\underbrace{g_{\mathrm{mt}}\left(\omega_{1},\Delta \omega ,k_{\mathrm{mt}},\delta \omega_{\mathrm{mt}},R_{2\mathrm{mt}}\right)}}, \end{array}$$

f is the pool concentration relative to water. Equation (8) will be basis of the linear least squares fitting approach described below.

## 3 METHODS

The core method for determining a solute spectrum can be divided into two steps: (1) using Eq. (2), the measured $R_{1}$, and magnetization $Z_{a}^{\mathrm{ss}}$ to solve for $R_{1\rho }$ at every irradiation power and offset; and (2) using Eq. (8) and a linear least squares fitting to determine the solute concentrations f at each solute offset and exchange rate. The linear least squares fitting in step 2 is often underdetermined and two constraints will be examined: non‐negative component amplitudes and non‐negative component amplitudes combined with L1 minimization, a form of least absolute shrinkage and selection operator (Lasso^26^) regression.

**FIGURE 1:**
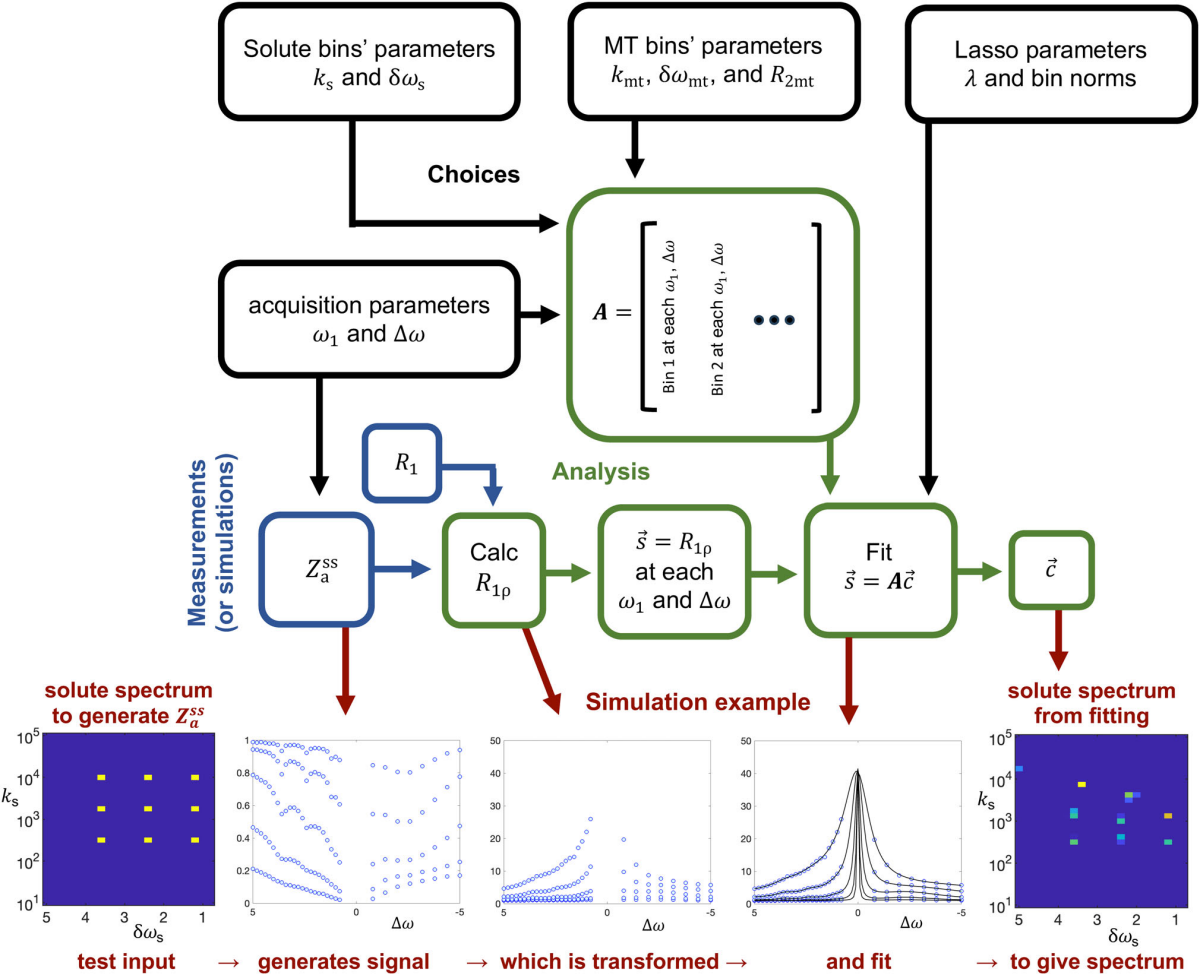
Diagram illustrating steps to generate a CEST solute spectrum. Black rectangles indicate acquisition and analysis choices. Blue rectangles indicate measurement or simulation results. Green rectangles indicate analysis steps. Red arrows show an example calculation. The example input is a simulation using a spectrum of nine solutes, each with a unique resonant frequency ($\delta \omega_{s}$) and exchange rate ($k_{s}$). The blue square on the left plots this spectrum, with color indicating solute concentration. The spectrum generates $Z_{a}^{\mathrm{ss}}$ (the water z‐magnetization) for a range of irradiation offsets and powers. This magnetization is then inverted to calculate $R_{1\rho }$ values, which are linearly fit to determine the bin weights $\vec{c}$, which are plotted as a solute spectrum on the right. Note that this fitted spectrum is qualitatively similar to the input spectrum, although with distortions.

Figure 1 illustrates how the solute spectrum is generated and plotted. Broadly speaking, there are three steps: choices, measurements, and analyses. The choices (black in Figure 1) include acquisition parameters ($\omega_{1},∆\omega$), solute bin parameters (k, δω), MT bin parameters (k, δω, $R_{2}$), and, when applying L1 minimization, Lasso fitting parameters (bin normalizations and λ, which is the relative weighting of the L1 minimization). The measurements or simulations (blue in Figure 1) give $Z_{a}^{\mathrm{ss}}$ and $R_{1}$. The analysis (green in Figure 1) solves for the weighting of all the bins ($\vec{c}$), with the solute spectrum defined as the weighting of solute bins.

The red arrows in Figure 1 illustrate the process using an example simulation. First, note that a solute spectrum (blue square) is defined as a 2D map of solute concentration as a function of $\delta \omega_{s}$ and $k_{s}$. Strictly speaking, it is a map of labile solute proton concentrations and should be termed a labile solute proton offset‐and‐exchange‐rate spectrum, but “solute spectrum” is used as a shorter nomenclature. This solute distribution (plus the macromolecular and water pool parameters) is the input data that drives the simulation of $Z_{a}^{\mathrm{ss}}$. The magnetization is then transformed and fit to give an output spectrum. The input spectrum generates the blue dots; the fitted spectrum (i.e., the output) comes from the fit lines.

Specifically, the solute spectrum comes from the vector $\vec{c}$ that solves the equation:

(9) $$\vec{s}=A\vec{c}.$$

The signal $\vec{s}$ is a vector of $R_{1\rho }$ values that come from inverting the measured $Z_{a}^{\mathrm{ss}}$ using Eq. (2). $\vec{s}$ is of length $n_{\mathrm{acq}}$ (= the number irradiation power/offset combinations used when acquiring the CEST data). ***A*** is a matrix with $n_{\mathrm{acq}}$ rows and $n_{\text{bins}}$ columns, where $n_{\text{bins}}$ is the number of possible components or “bins” used to fit the signal. Each bin corresponds to a function in Eq. (8), with weighting of this function determined by the corresponding element in $\vec{c}$. $\vec{c}$ is a vector of pool concentrations (f), scaled by function normalization related to Lasso fitting (discussed below). There are three types of bins: (1) two bins for the direct effect; (2) bins for possible solute contributions (with each bin having a unique set of k and δω values defining a particular solute); and (3) bins for one or more MT pool contributions (with each bin having a unique set of k, δω, and $R_{2}$ values). All $\vec{c}$ values are determined concurrently by solving for the $\vec{c}$ that:

(10) $$\text{minimizes}\;{\left\|A\vec{c}-\vec{s}\right\|}^{2}+\lambda \sum_{i=1}^{n_{\text{bins}}}c_{i},\text{subject to}\;c_{i}\geq 0\;\text{for}\;i=1,\cdots ,n_{\text{bins}},$$

where λ = 0 for the purely non‐negative case without Lasso minimization. To compensate for the inversion of the measured signal in Eq. (2) (which is the essential step that makes the fit linear in solute contributions), the fit is weighted by $Z_{a}^{\mathrm{ss}}$. For instance, both signals in $\vec{s}$ and corresponding rows in ***A*** are weighted by the measured $Z_{a}^{\mathrm{ss}}$ when fitting for $\vec{c}$.

For this proof‐of‐concept paper, we did not optimize the acquisition parameters ($\omega_{1},∆\omega$) or bin parameters (k, δω, $R_{2}$). The values are given in the Appendix A and were chosen following a few general guidelines. Irradiation amplitudes $\omega_{1}$ were chosen to be viable on an animal imaging system (0.5–11.4 μT, in simulations, and 0.57–11.7 μT in experiments). Irradiation offsets ∆ω were chosen to capture the range of offsets of CEST solutes and exchanging macromolecules (−5 and +5 ppm from the water resonance). Offsets between −0.8 and +0.8 ppm were excluded because of the difficulty reliably applying Eq. (2) when the measured $Z_{a}^{\mathrm{ss}}$ is small and $\cos^{2}\theta$ is equal or close to zero. Bin parameters were chosen with minimal modeling assumptions. For bins designed to characterize solutes, δω was limited to positive values (to match known solute offsets, e.g., Khlebnikov et al^16^) and chosen to match the acquisition ∆ω values (to avoid spurious fitting of solutes with narrow peaks between data points). Because there are indications that exchanging macromolecules have both negative and positive offsets (e.g., Zaiss et al^27^) and because their large $R_{2}$ values create large peak widths, MT bins were assigned δω values spanning the full −5 to 5 ppm range and were not required to match the acquisition ∆ω. Additionally, the MT bins include a $R_{2}$ dimension that spans both MT and NOE phenomenon (500–50 000 1/s). Finally, simulations and experiments were performed at 15.2 T and simulations used a SNR of 500, except where noted.

Two least squares fitting methods were used to solve for $\vec{c}$ in Eq. (10): non‐negative least squares minimization of $\vec{s}-A\vec{c}$ (using the lsqnonneg function in MATLAB 2024b) and non‐negative least squares minimization of $\vec{s}-A\vec{c}$ with L1 minimization of $\vec{c}$ (using the MATLAB implementation l1_ls_nonneg.m by the Boyd lab at http://www.stanford.edu/∼boyd/l1_ls/ ^28^ and sometimes called Lasso). Note that the current approach should not be confused with a previous use of Lasso minimization applied to CEST imaging.^29^ In this previous work, $\vec{c}$ corresponded to Z‐spectra and $\vec{s}$ to desired imaging metrics, such as conventional amide proton transfer (APT) Lorentzian fits. The current work applies Lasso minimization to a different problem, where $\vec{c}$ is the weightings of many possible contributing solutes, macromolecules, and direct effects, and $\vec{s}$ is the resulting $R_{1\rho }$ for each acquisition. The idea in the current work is similar to the signal decomposition seen in the multi‐exponential T_2_ (MET2) literature,^30^ where the measured signal is decomposed into many contributing T_2_ bins via a non‐negative linear least squares fitting. (MET2 fittings do not typically use L1 minimization and instead often regularize to produce the smoothly varying component weights expected for relaxation).

Adding the L1 minimization to the underdetermined fittings in the current work leads to sparser solutions (i.e., more elements with $\vec{c}$ = 0), which may be advantageous when fitting the expected discrete distribution of exchanging site offsets and exchange rates, especially if there are broad MT pools contributing to signals at many offsets. The L1 minimization requires setting the relative weighting (λ) of the L1 minimization of $\vec{c}$ versus the L2 minimization of $\vec{s}-A\vec{c}$, along with normalization factors of the bin functions (gs in Eq. [8]). λ = 0.005 was chosen empirically to give reasonably accurate results in simulations. Bin normalization values were chosen to roughly match expected values, which were 0.001 for solute bins (matching expected concentration relative to water), 0.1 for MT bins, 25 or 10 1/s for the $\sin^{2}$ bin (roughly matching expected or measured $R_{2}$ values in the simulations or experiments, respectively), and 1 1/s for the $\cos^{2}$ bin (matching the expected $R_{1}$ value). These bin normalizations are relevant only because we are doing a Lasso minimization that includes different types of bins. The fitting methods were tested in simulations where $Z_{a}^{\mathrm{ss}}$ was calculated as the steady state solution to the coupled Bloch‐McConnell equations, and in experiments where $Z_{a}^{\mathrm{ss}}$ was directly measured. All simulations and data fittings were implemented in MATLAB 2024b. Simulation code is available at https://github.com/gochberg/solute_spectrum.

CEST simulations were performed for seven digital phantoms referred to as “diagonal 2,” “square 4,” “square 9,” “square 9 + MT,” “extreme square 9+MT,” “δω separation,” and “k separation.” Diagonal 2 has two solutes with $k_{s}$ = 316 and 10 000 1/s and $\delta \omega_{s}$ = 1.2 and 3.6 ppm, respectively. Square 4 is like diagonal 2, but with all four possible $k_{s}$ and $\delta \omega_{s}$ combinations. Square 9 has nine solutes with $k_{s}$ = 316, 1778, and 10 000 1/s and $\delta \omega_{s}$ = 1.2, 2.4, and 3.6 ppm. Square 9 + MT is identical to square 9, but with the addition of an MT pool with $R_{2\mathrm{mt}}$ = 6000 1/s, $k_{\mathrm{mt}}$ = 49 1/s and $\delta \omega_{\mathrm{mt}}$ = −2.35 ppm.^31^ Extreme square 9 + MT is similar but with a wider spread in exchange rates: $k_{s}$ = 31.6, 1778, and 31 623 1/s. “δω separation” has $\delta \omega_{s}$ = 2.6, 3.4, 4.0 and 4.4 ppm and $k_{s}$ = 1000 1/s; “k separation” has $k_{s}$ = 316, 1000, and 5623 1/s and $\delta \omega_{s}$ = 3.0 ppm. Each digital phantom was designed to test the ability to distinguish overlapping signals. For each of the “square” phantoms, at every solute resonant frequency there are multiple exchange rates, and at every exchange rate there are multiple resonant frequencies. The two “separation” phantoms examine each dimension independently and at various resolutions.

Data was acquired on a 15.2 T Bruker Biospec Avance III scanner using a single‐shot RARE readout after 8 s CEST irradiation. Measurements were made on four phantoms of known chemical composition: phosphocreatine (PCr), PCr + myoinositol (MI), PCr + MI + γ‐aminobutyric acid (GABA), PCr + MI + GABA + creatine (Cr). Phantom details are given in the Appendix A, as are CEST measurement and analysis parameters (which are near identical to those used in the simulations). In addition, an inversion recovery sequence quantified $R_{1}$ and a WASABI^32^ sequence was used to measure and correct for spatial variations in $B_{0}$ and $B_{1}$; a separate measure of steady‐state saturation and relaxation rates was made in an MnCl_2_ phantom (with no exchanging solutes) to calibrate differences in $B_{1}$ as measured using short pulses (as in WASABI) and applied as long pulses (as in CEST).

The CEST measurements of the chemical phantoms were used to generate solute spectra. Additionally, as a point of comparison, solute spectra were generated using the results of conventional non‐linear least squares fits of the solute offsets and exchange rates. These fits to the steady‐state solution of the coupled Bloch‐McConnel equations serve as a gold standard in this study. However, unlike the solute spectra generated from linear fits, they required a priori knowledge of the number of exchanging sites and their chemical concentrations, along with consistency constraints on the fitted offsets and exchange rates. That is, the PCr offsets and exchange rates found when fitting the PCr phantom were then used as constraints on the PCr peak when fitting the PCr + MI phantom, and the resulting fitted MI offset and exchange rate was used as additional constraints when fitting the PCr + MI + GABA phantom, and so on.

Additionally, although the CEST simulations were designed to match viable experiments on an animal system, and the CEST measurements applied this approach to chemical phantoms with four solutes (PCr, MI, GABA, and Cr), a single ambitious $R_{1\rho }$ simulation was run to explore what would be necessary to resolve a more realistic in vivo case of nine neuronal solutes (glucose [Glc], MI, Cr, PCr, GABA, taurine [Tau], glutamate [Glu], glutamine, and amides) with 16 labile sites. Using $R_{1\rho }$ avoids any issues from Eq. (2)'s approximations or singularities near the water resonance. Instead, no conversion from $Z_{a}^{\mathrm{ss}}$ is necessary and the same function (Eq. 5) is used to both generate and fit the data (after adding noise). The fit used four times as many data points (as detailed in the Appendix A and consisting of 60 irradiation frequencies, including at and near the water resonance, and 10 irradiation powers) and 1.9 times as many fit bins (including coverage of the solute offsets). The goal of this last simulation was to stretch reasonable limits to see what is possible and thereby provides a target for later optimizations of the acquisition and analysis protocols, including improvements in pulse sequences and fitting constraints.

## 4 RESULTS

**FIGURE 2:**
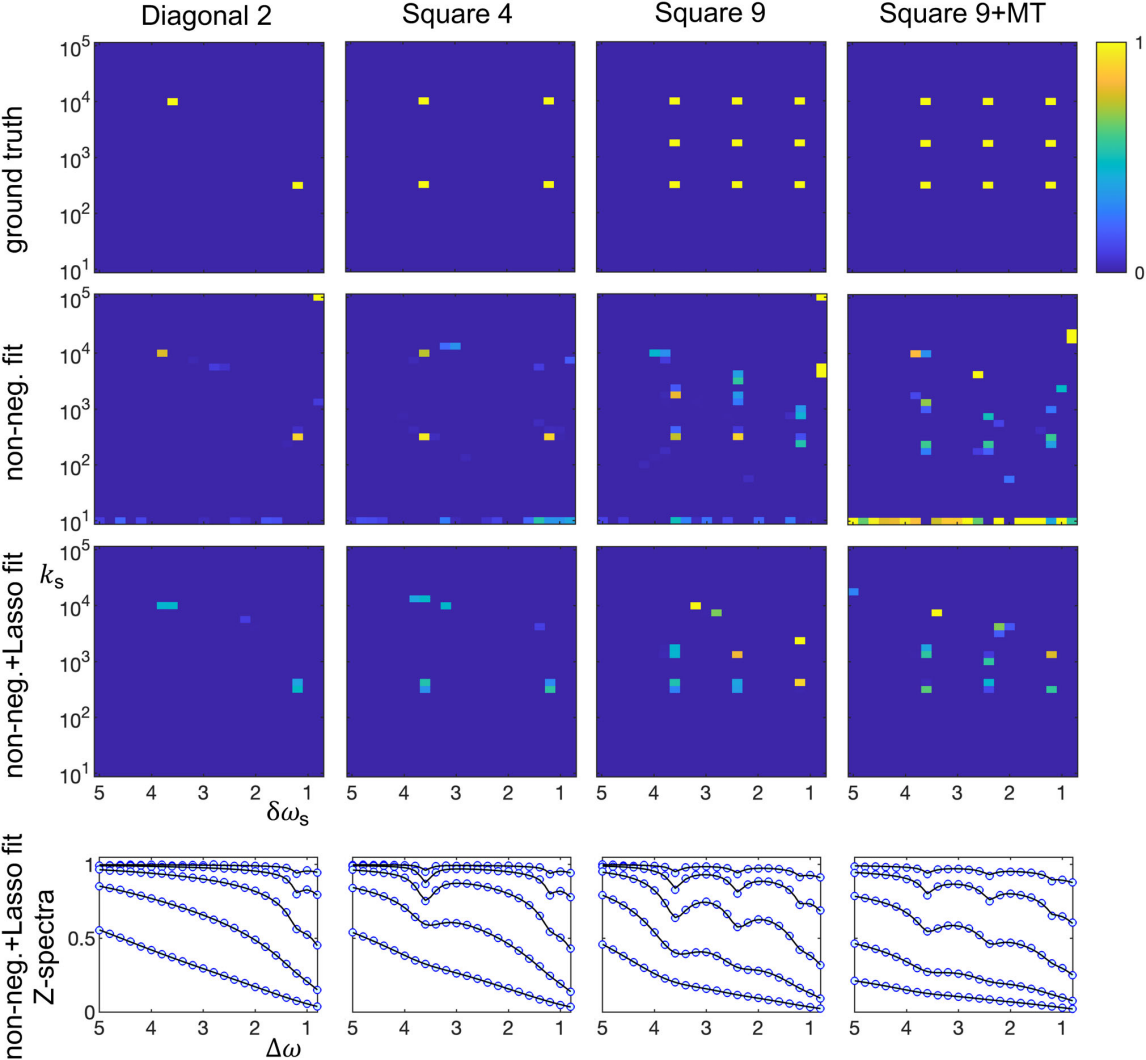
Fitted solute spectra of four simulated digital phantoms: diagonal 2, square 4, square 9, and square 9 + MT. The solute spectra are displayed as 2D maps of fitted concentration at each solute offset $\delta \omega_{s}$ (ppm) and exchange rate $k_{s}$ (1/s). First row: the “ground truth” plots show the underlying data used by the simulations, with 100% of a solute's concentration in the bin with the closest offset/exchange rate. Second row: solute spectra from linear fits using non‐negative constraints. Third row: solute spectra from linear fits using non‐negative constraints plus Lasso L1 minimization. Fourth row: simulated Z‐spectra (blue dots generated by first row spectra) and fit lines (corresponding to third row fitted spectra). Key results: (1) simulated CEST Z‐spectra can be fit using an under‐determined linear approach to give a very rough qualitative indication of the number, offsets, and exchange rates of the contributing solutes without a priori assumptions; (2) in contrast, visual inspection of Z‐spectra (i.e., the fourth row) gives no obvious indication of the exchange rates or number of solutes at each frequency; (3) a non‐negative constraint produces more spurious solutes, including many at the smallest exchange rate. Adding the L1 minimization of the Lasso approach reduces spurious solute fits. To facilitate comparisons, the colormap of all figures is limited to 0 to 1 (in units of 0.001 of the water concentration), with greater bin values mapped to 1. Lasso, least absolute shrinkage and selection operator; MT, magnetization transfer.

**FIGURE 3:**
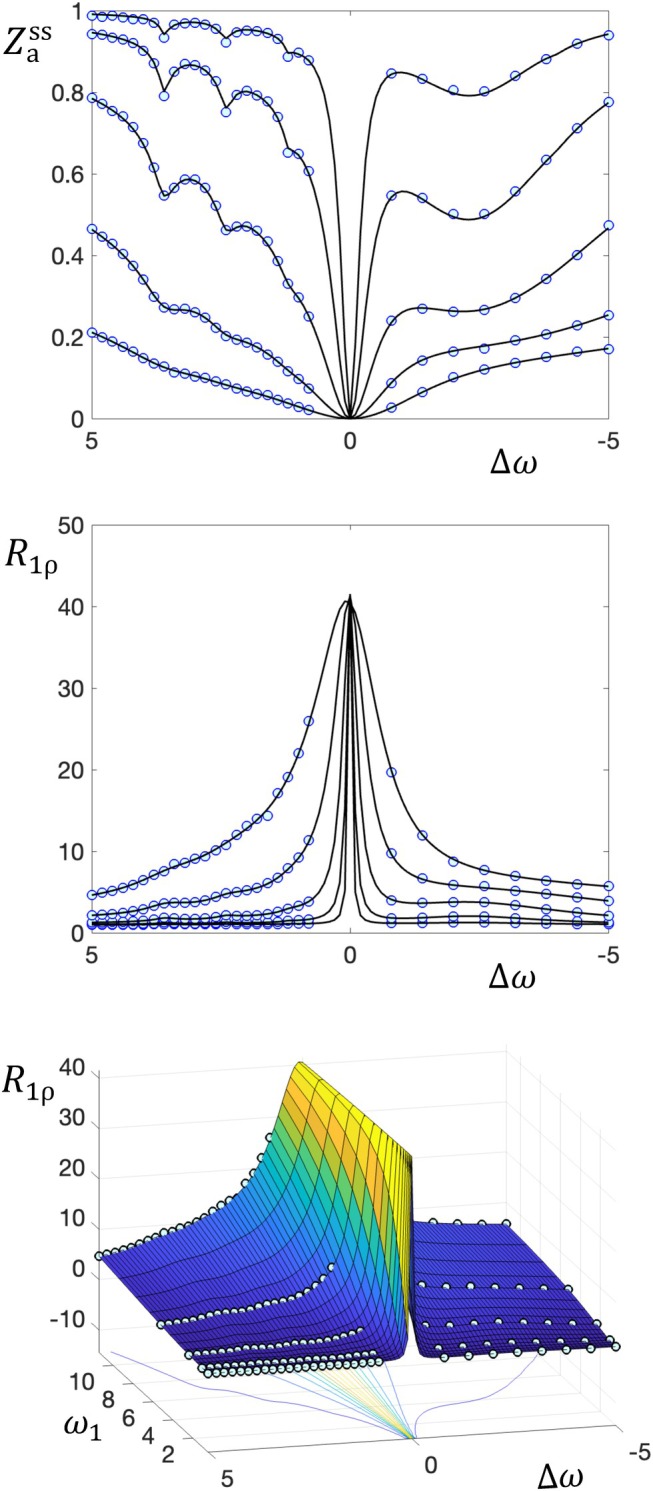
Simulated square 9 + MT data (blue circles) and non‐negative Lasso fits, plotted three ways. Top: $Z_{a}^{\mathrm{ss}}$ versus the acquisition offset ∆ω (ppm) for multiple acquisition powers $\omega_{1}$ (μT), along with fit lines. Middle: $R_{1\rho }$ (1/s) versus ∆ω for multiple $\omega_{1}$, along with fit lines. Bottom: the same $R_{1\rho }$ data, but this time as a 3D plot with a fit surface, which better matches the approach in the current work. Lasso, least absolute shrinkage and selection operator; MT, magnetization transfer.

**FIGURE 4:**
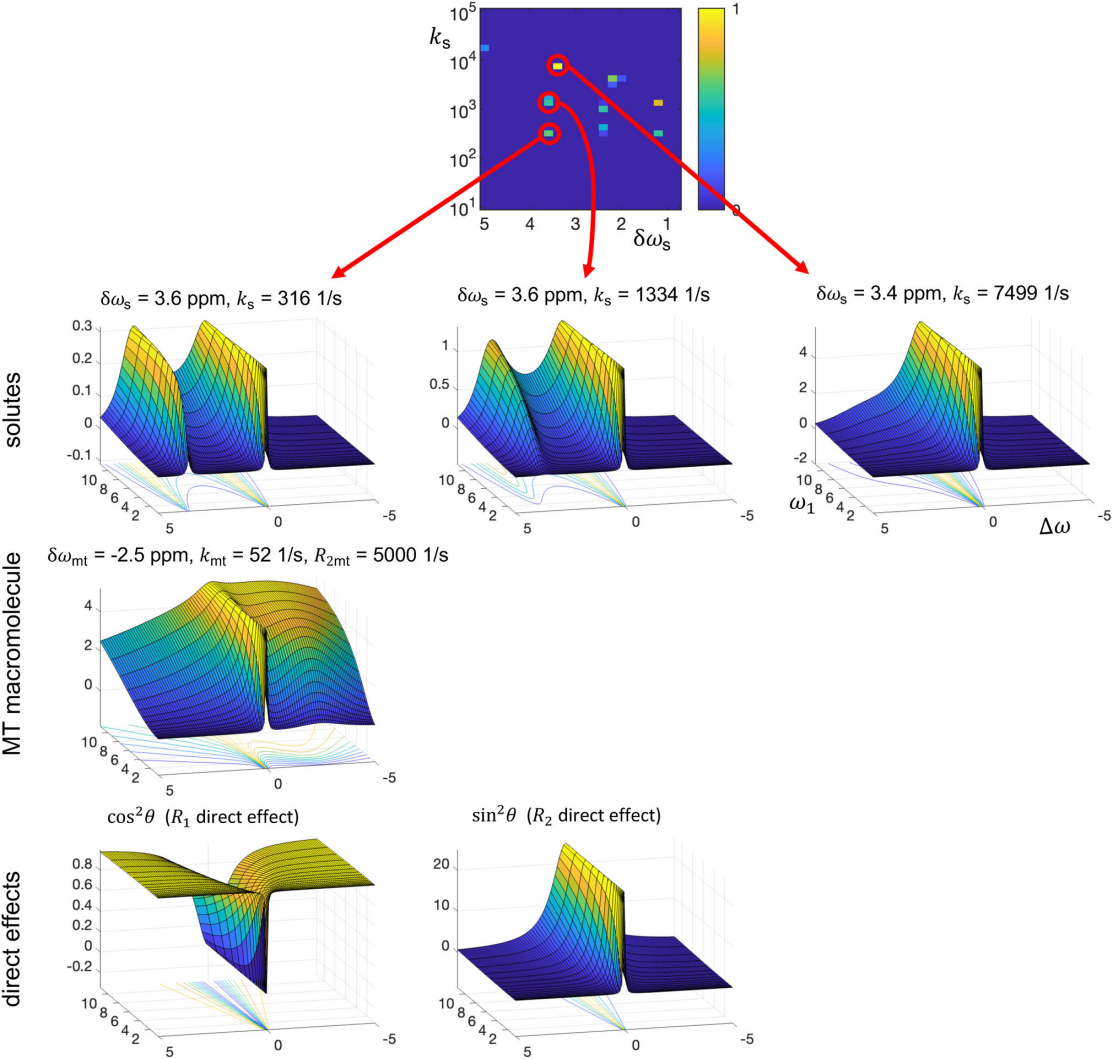
Illustration of the connection between the square 9 + MT solute spectrum and the components of the fit surface. The $R_{1\rho }$ fit surface in Figure 3 is a linear sum of multiple components, each of which is a different function of ∆ω (ppm) and $\omega_{1}$ (μT); the solute components correspond to points in the non‐negative + Lasso spectrum (which is the bottom‐right spectrum in Figure 2). Six example components are plotted, including three from solutes of increasing exchange rates (red arrows), one from an MT macromolecular pool, and two from direct effects. These six example surfaces are taken from the 18 fit components greater than a 0.1 threshold, of which 13 come from solutes, three from macromolecules, and two from direct effects. Lasso, least absolute shrinkage and selection operator; MT, magnetization transfer.

**FIGURE 5:**
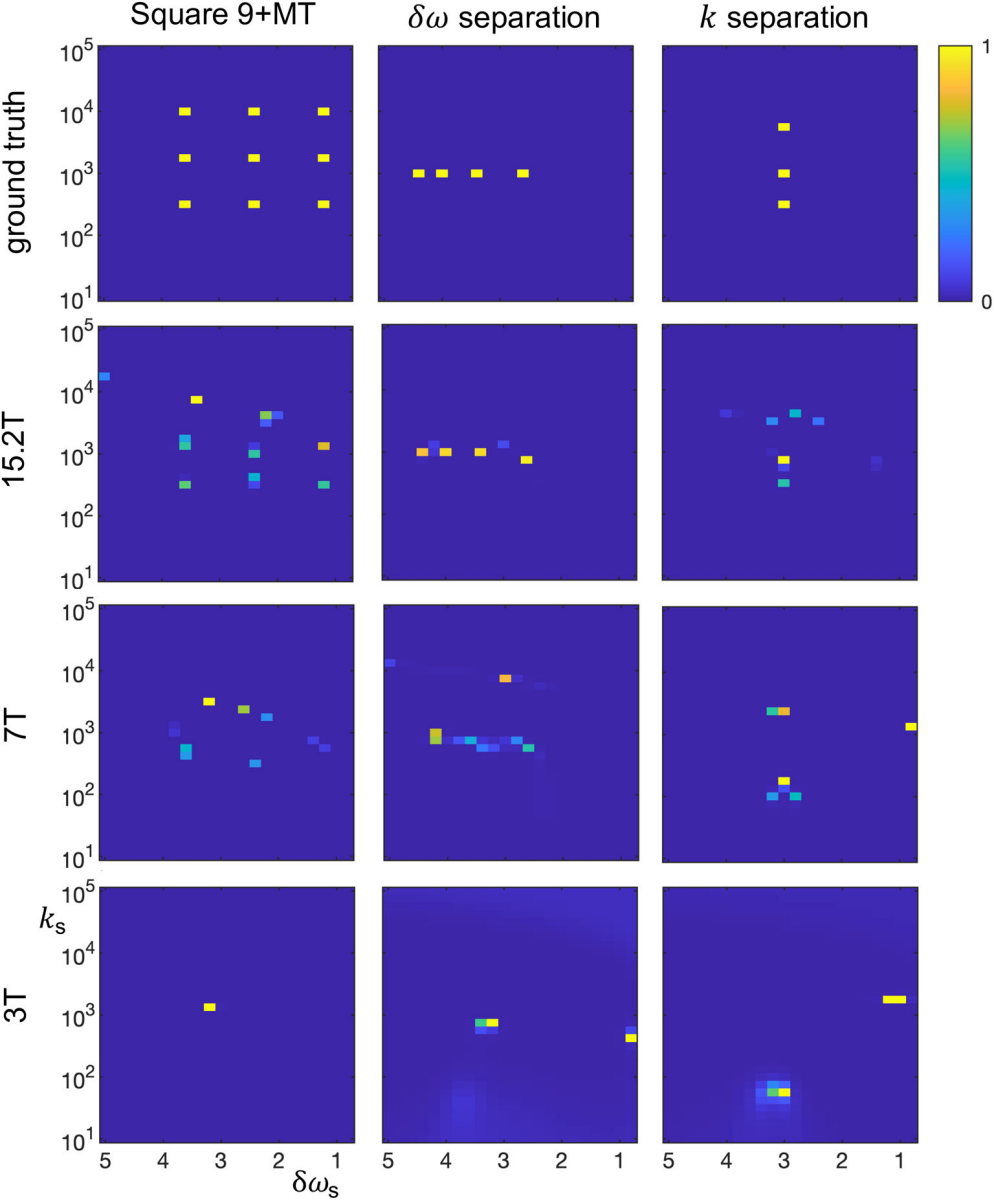
The dependence on static field strength B_0_ of non‐negative + Lasso fits of the simulated signal for three digital phantoms: square 9 + MT, δω separation, and k separation. Relaxation rates, SNR, and frequency offset in ppm are held constant; hence, frequency offsets in 1/s scale with the static field strength, causing fits to become more difficult at lower field strengths. Specifically, at lower field strengths, the shape of the square 9 + MT phantom is distorted and the effective resolution of the separation phantoms degrades. Lasso, least absolute shrinkage and selection operator; MT, magnetization transfer.

**FIGURE 6:**
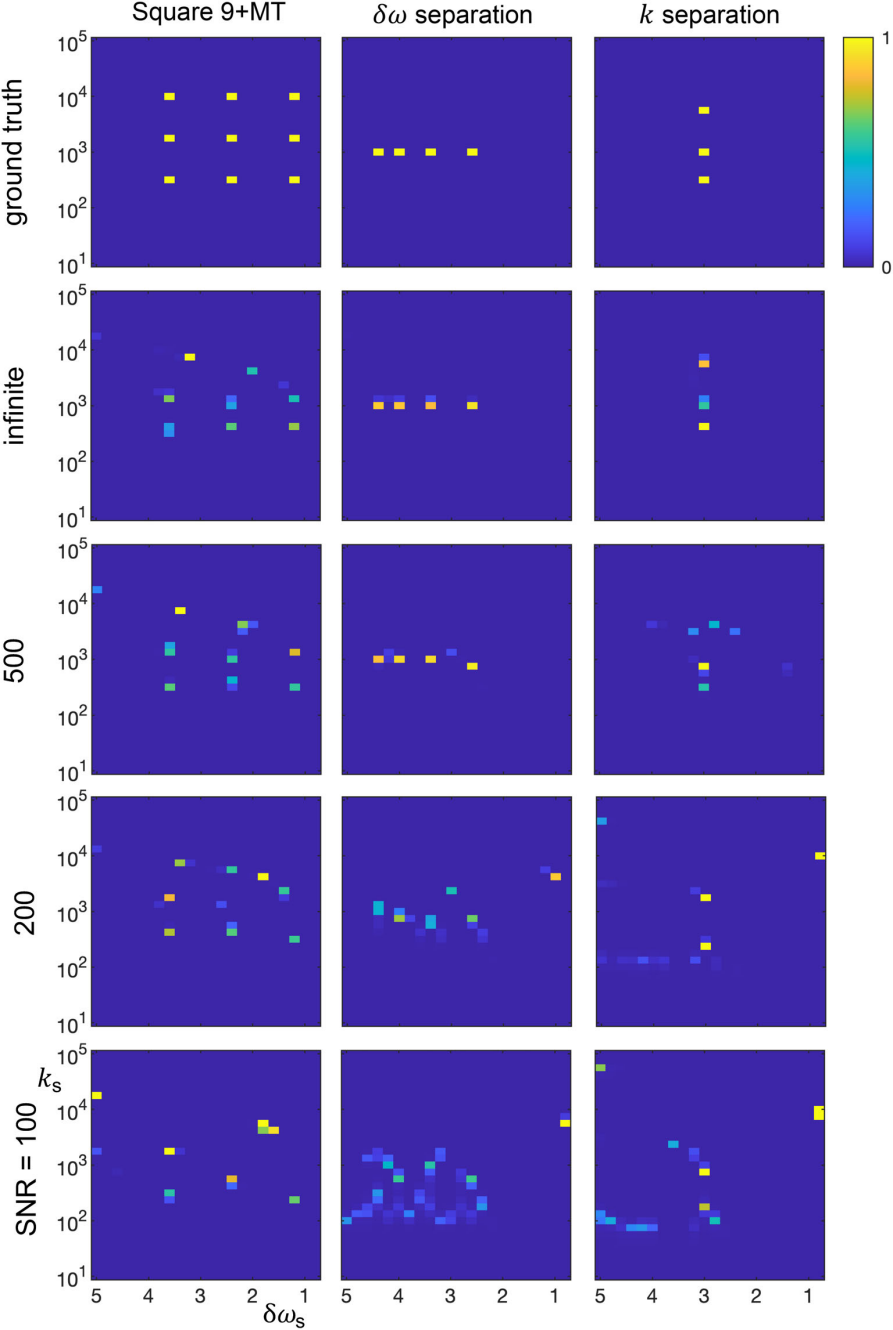
The dependence on SNR of non‐negative + Lasso fits of the simulated signal for three digital phantoms: square 9 + MT, δω separation, and k separation. At lower SNR values, the shape of the square 9 + MT phantom is distorted and the effective resolution of the separation phantoms degrades Guided by these results, all simulations used SNR = 500. Lasso, least absolute shrinkage and selection operator; MT, magnetization transfer.

**FIGURE 7:**
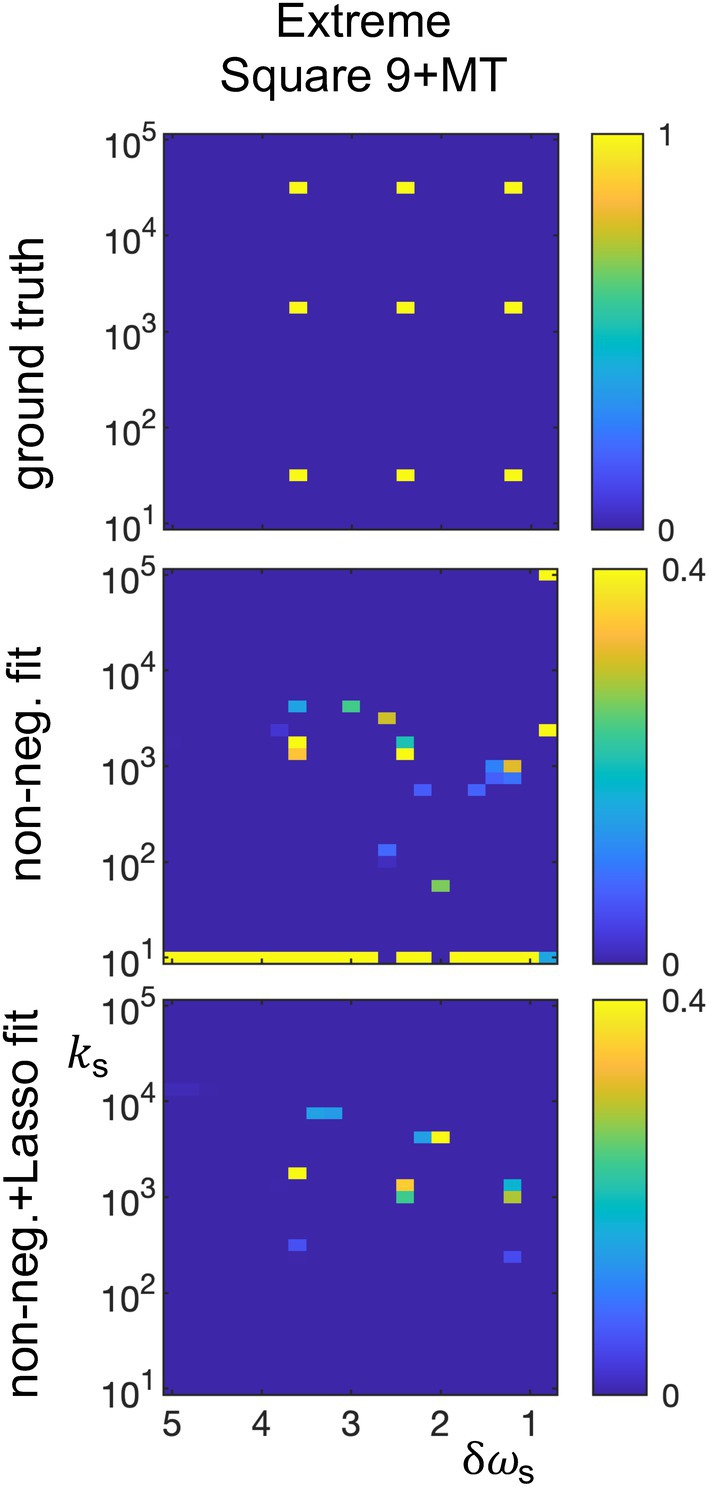
Fits of the simulated digital phantom extreme square 9 + MT, illustrating limitations when fitting groups of solutes that include some with very fast and very slow exchange. The colormap of the fits has been scaled to 0.4. MT, magnetization transfer.

**FIGURE 8:**
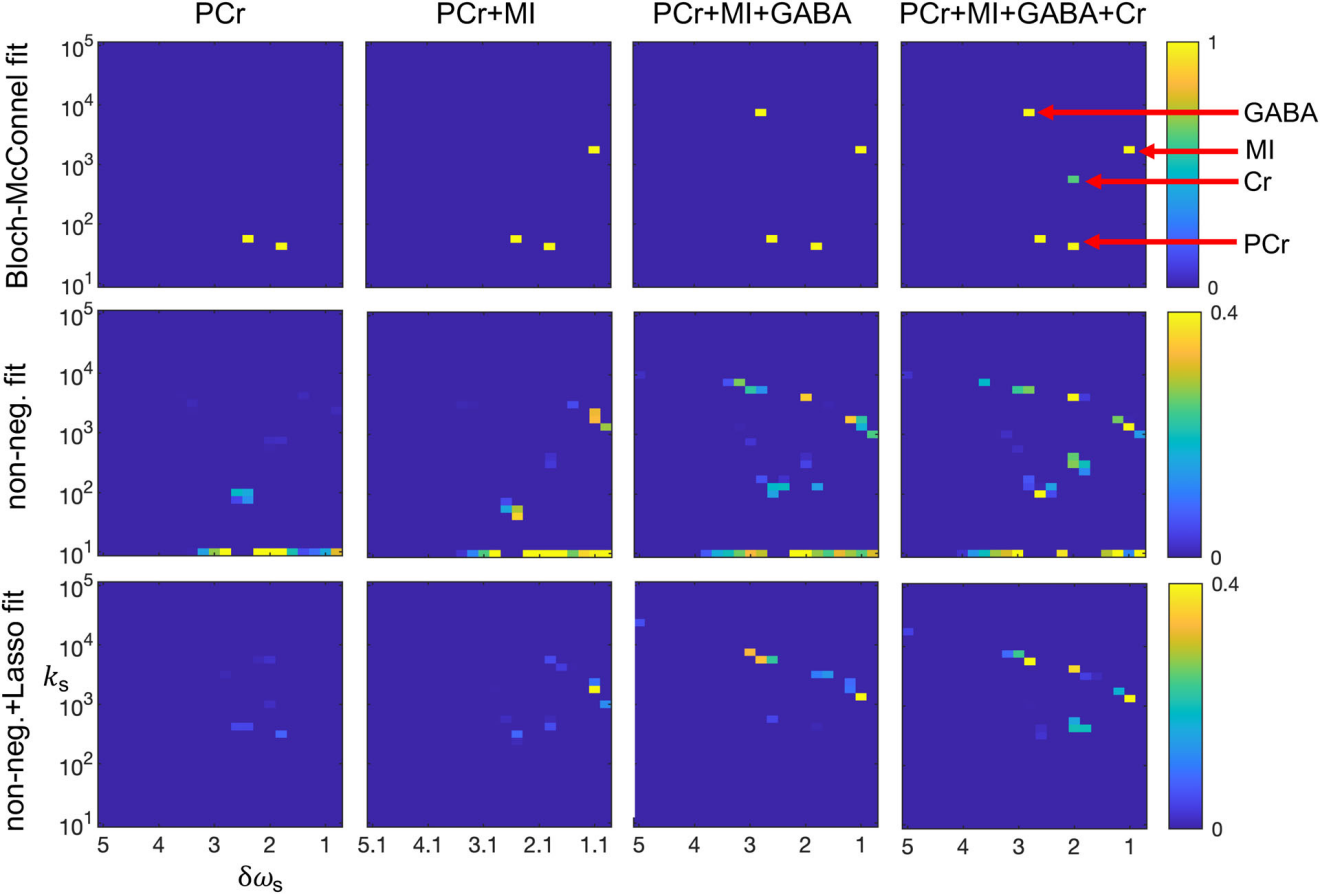
Fits of four chemical phantoms. The Bloch‐McConnel fit (first row) is treated as the gold standard here. Note the ability of both linear fits to give rough qualitative matches to the Bloch‐McConnel non‐linear fits that rely on a priori information, although with clear distortions and spurious results. The non‐negative fit does a superior job by roughly matching all solutes, although with a biased and blurred characterization of the slow exchanging PCr sites and spurious peaks, especially approximately 2 ppm and 4000 1/s. The non‐negative + Lasso fit produces extremely low amplitude PCr peaks, likely because of its limited sensitivity (in its current implementation) to slow exchanging solutes. The colormap of the fits has been scaled to 0.4. Lasso, least absolute shrinkage and selection operator; PCr, phosphocreatine.

**FIGURE 9:**
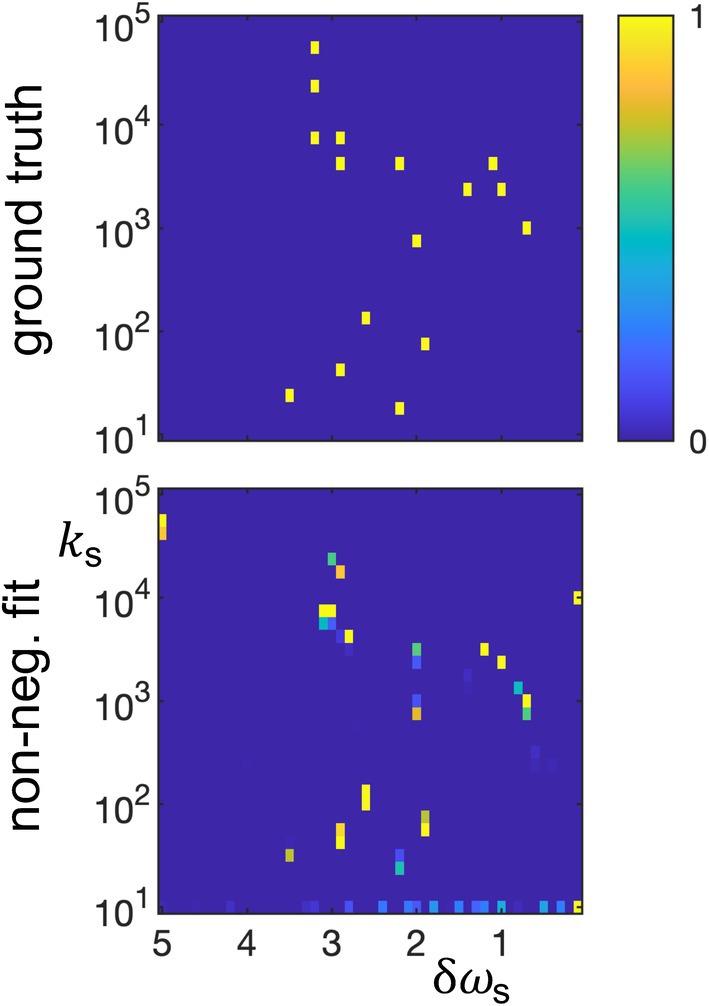
Fitted solute spectrum of the simulated signal for nine neuronal metabolites with 16 labile sites.

Broadly speaking, the results are organized into five categories: validation tests using simulated digital phantoms with varied solute distributions in δω‐k‐space (Figure 2), interpretation of solute spectra (Figures 3 and 4), examination of fitting resolution and range of applicability (Figures 5, 6, 7), validation tests using experimental phantoms (Figure 8), and a simulation that would be experimentally unrealistic, but can guide future development and application (Figure 9).

Figure 2 shows the most important simulation results of the paper. Four digital phantoms (shown as “ground truth” solute spectra) are used to simulate CEST Z‐spectra (bottom row), which are then fit using non‐negative and non‐negative + Lasso constraints (with the resulting fitted spectra in the 2nd and 3rd rows). Note the key result: although the fittings have clear spurious components and distortions, they nonetheless are rough qualitative matches of the number, offsets, and exchange rates of the solutes in the underlying data. That is, they roughly reproduce the solute distributions in δω‐k‐space. This signal inversion is achieved without using any a priori information. This fitting result holds with and without a broad MT pool and with multiple pools at the same offset and multiple pools at the same exchange rate. In contrast, the conventional Z‐spectra (bottom row) indicate the resonant frequencies of the solutes, but gives no obvious indication of the exchange rates or number of solutes at each frequency.

Figure 2 also allows comparison of non‐negative versus non‐negative + Lasso fittings. First, noise shows up in non‐negative fittings as contributions from bins with minimal exchange rates. This separation of noise is advantageous, because in most cases it is distinguishable from physically meaningful contributing solutes. Second, and more detrimentally, non‐negative fittings produce erroneous contributions from many solute bins, especially when a broad MT pool is present. In contrast, Lasso's L1 minimization favors sparse solutions.

Figures 3 and 4 illustrate the connection between fitting CEST data and the resulting solute spectrum. Figure 3 plots the simulated data and fit for the square 9 + MT digital phantom in three ways. The top sub‐figure plots $Z_{a}^{\mathrm{ss}}$ versus ∆ω for multiple $\omega_{1}$ values, which matches the typical format in the CEST literature and matches the bottom right sub‐figure in Figure 2, but now over the full range of offsets. The middle sub‐figure inverts $Z_{a}^{\mathrm{ss}}$ to plot $R_{1\rho }$ versus ∆ω for multiple $\omega_{1}$ values, which matches the key aspect of the fitting that solutes contribute linearly to the y‐axis metric. The bottom sub‐figure replots $R_{1\rho }$, but as a 2D plot versus both ∆ω and $\omega_{1}$, with the fit as a 2D surface and with a corresponding contour plot underneath. This 2D surface more clearly displays the dependence on $\omega_{1}$ and can be more clearly connected to the corresponding solute spectrum. Figure 4 makes this connection by plotting the 2D surfaces that are fit contributions from individual bins. It shows six such surfaces, three from solutes, one from MT, and the two direct effect bins. Note how the shape of each component depends on the corresponding solute offset and exchange rate.

As noted above, the acquisition and analysis parameters have not been optimized, and the highest possible sensitivity as a function of the sample parameters is unknown. Additionally, we expect that a full sensitivity analysis will show a complex co‐dependence on many factors, including the total number of contributing solutes and their distribution in δω‐k‐space. Nonetheless, we can get some feel for the sensitivity and effective resolution of the current implementation. Figures 5 and 6 show the field and SNR dependence when fitting the square 9 + MT, δω separation, and k separation phantoms using non‐negative + Lasso constraints. These results motivate using 15 T and SNR = 500 in other simulations, as they produce rough qualitative reproduction of the square 9 + MT digital phantom and sub‐ppm and sub‐kHz resolutions in the “separation” phantoms. However, the solute position in δω‐k‐space matters, and Figure 7 examines the sensitivity when there are solutes with very fast and very slow exchange. Note the increased discrepancy of the fits versus the underlying data in comparison to the square 9 + MT results in Figure 2, and especially note the limited sensitivity of the Lasso fit to very slow exchange. This limit in regime is relevant to application of the current protocol in practice, as some relevant endogenous solutes exchange slowly.

Figure 8 shows the fitted solute spectra of four chemical phantoms of increasing complexity. The key result is that the linear fits give a (very rough) qualitative match to the conventional Bloch‐McConnell fits (which leveraged a priori knowledge of chemical composition). Note the greater sensitivity of the non‐negative fit to the two slow exchanging PCr sites in comparison to the non‐negative + Lasso fit, although it is unable to separately resolve the two slow exchanging sites. (This increased sensitivity is consistent with the simulation results in Figure 7). Note also that both linear fits include spurious peaks (e.g., near 2 ppm and 4000 1/s in the PCr + MI + GABA + Cr phantom).

Figure 9 illustrates a more complex (and more biologically realistic) fitting of nine neuronal solutes with 16 labile sites. The decomposition is qualitatively accurate (at least relative to previous CEST/ $R_{1\rho }$ analyses). However, to achieve realistic fittings, several relevant experimental and analysis roadblocks were ignored, as discussed below.

## 5 DISCUSSION

The goal of this work is to demonstrate proof of concept for generating solute spectra without a priori constraints on the exchange rates, offsets, or number of contributing solutes. The key CEST signal characteristic that makes this disentanglement possible is the linearity of the contributions of individual solutes to the water relaxation rate in the rotating frame, $R_{1\rho }$. This linearity is a general feature of systems where rates add in parallel (e.g., the rate of water filling a tub from multiple faucets). In the $R_{1\rho }$ literature, this linearity is long established (e.g., Trott and Palmer^33^) and has been leveraged to separate direct versus exchange contributions to CEST by calculating metrics or fittings proportional to $R_{1\rho }$ by inverting the measured signal (e.g., Zaiss and Bachert,^23^ Zaiss et al,^34^ Zu et al,^35^ Jin and Chung,^36^ and Xiao et al^37^). In this work, we extend this approach to separate, at least qualitatively, individual contributions from all solutes and macromolecules by using non‐negative and sparse constraints to the underdetermined linear fitting.

The choices made in this paper for acquisition and analysis parameters are reasonable for an animal imaging acquisition at high field, but very likely not optimum. Optimization would require understanding how the acquisition, analysis, and sample parameters dictate the sensitivities to solute number, concentrations, offsets, and exchange rates. These dependencies are complex and beyond the scope of the current work. However, it is still worth discussing one particularly important choice: the linear fitting method. Figure 2 shows how adding the L1 minimization in the Lasso fit can increase sparsity and effectively filter out spurious fit components. However, Lasso fits require assigning the relative weighting of L1 minimization and, if there are multiple types of bins, the bin normalization values. Hence, the Lasso fitting is not fully in the spirit of the paper's goal to minimize a priori assumptions when analyzing CEST data. Further, adding the L1 minimization appears to decrease sensitivity to slowly exchanging solutes, as indicated by Figures 7 and 8. Hence, using a solely non‐negative constraint is likely the better choice in most cases. However, like the choice of acquisition and analysis parameters, the choice of linear fitting method may have room for significant improvement.

Because tissues often have one or more macromolecular pools with large $R_{2}$ values, we included corresponding MT bins. These bins are described by analytic fitting functions with non‐zero $R_{2}$ and include a third sample parameter dimension ($R_{2}$) in addition to the two dimensions used in the solute bins (k and δω). For simplicity (and to maintain linearity), we ignored the scaling effect a large MT pool has on the interpretation of the fitted bin amplitudes.^38^ As this effect scales all solute bins uniformly, any resulting bias in the fitted solute concentrations relative to water will not affect the current goal of revealing the number, exchange rates, offsets, and relative concentrations of contributing solutes. Additionally, following,^38^ the $R_{1}$ used in Eq. (1) should in practice be the measured value (which includes MT contributions). Note also that we are not accounting for non‐Lorentzian macromolecular lineshapes because: (1) within the relatively narrow 10 ppm range of offsets where CEST solute pools reside, a Lorentzian lineshape is assumed good enough and simpler; (2) our fit approach will allow for the sum of multiple Lorentzians of different widths and offsets, which may be able to account for any non‐Lorentzian features; (3) the macromolecular lineshape at small offsets is not known; and (4) the super‐Lorentzian lineshape typically used in vivo at large offsets has a singularity near the zero offset, which is not physical.

Like conventional NMR spectra, CEST solute spectra are a model‐free description of the contributing solutes and are generated from measured magnetization. However, NMR spectroscopy uses a complete set of orthogonal Fourier components to decompose the measured solute magnetization. No information is lost in generating the spectra and the transformation is reversible by the inverse Fourier transform. In contrast, a CEST solute spectra is an under‐determined linear fitting of an indirect measure of solute magnetization with a large solution space effectively requiring non‐negative constraints and (in the case of Lasso fitting) L1 minimization. This fitting process is more like that seen in MET2 than localized NMR spectroscopy.

The similarities in MET2 and CEST solute spectrum fitting processes hint at possible similarities in applications and challenges. MET2 gives novel tissue characterizations and guidance for separate analyses. MET2 generated T_2_‐spectra has, for example, been directly used to characterize white matter myelin content.^30^ It also has been useful as an exploratory tool, providing guidance, for example, for developing clinically viable methods to assess bone composition.^39^, ^40^, ^41^ Solute spectra may have similar utility, and there are several areas where decomposition of the contributors to the CEST signal would be useful. For example, the protein/peptide amide peak^42^ is typically modeled as a single exchanging pool, but likely has contributions from many distinct components such as carnosine, anserine, and homocarnosine.^12^ Similarly, contributions from NOE up field from the water resonance are typically modeled as one or two^3^, ^43^, ^44^, ^45^ lipid sites, but contain many underlying distinct resonances.^46^, ^47^ Additionally, being able to disentangle the contributors to the CEST signal along two dimensions (solute resonate frequency offset and exchange rate) may enable the detection of CEST resonances that are difficult to isolate (e.g., serotonin^48^ or any of the individual metabolites that can be measured spectroscopically from cell extracts).^19^ Of course, any eventual benefits of this new approach are dependent on the robustness and sensitivity in practice, which is unknown. The current proof‐of‐concept study used long acquisitions and high field strength and SNR. For practical use, further development of acquisition, analysis, and fitting approaches is needed. Additional issues include motion effects, field inhomogeneities, and the absence of a gold‐standard for validation in tissue. Finally, interpreting solute spectra will be essential and non‐trivial, as was the case for comparable MET2 development.^30^, ^49^

The current initial results are mixed. Using a reasonable acquisition for a high‐field animal research study, Figure 2 shows an ability to qualitatively characterize the distribution of exchanging solutes in δω‐k‐space, something not possible from simple inspection of the Z‐spectrum, but with clear distortions and spurious components. Figures 5 and 6 suggests a resolution on the scale of 0.4 ppm and 100's of 1/s. However, Figure 7 indicates that this resolution may degrade when there are very fast or slow exchanging solutes, an example of the unknown resolution dependency on the complete set of solute, acquisition, and analysis parameters. Figure 8 indicates a rough qualitative characterization when fitting experimental data from four solutes (with 5 labile sites), but again with clear deficiencies. Figure 9 takes a different approach: instead of basing the simulation on a viable CEST animal research protocol, it examines what is possible if we ignore multiple issues to gain insight into potential future post‐optimization applications. By taking four times as many data points, ignoring experimental difficulties in measuring $R_{1\rho }$ directly, assuming accurate signal modeling, and increasing acquisition and bin sampling (to partially bypass the complexities of relative acquisition sampling, bin sampling, and solute distributions), Figure 9 shows promising characterization of a more realistic neuronal digital phantom with 16 exchanging sites.

## Data Availability

Simulation code is available at https://github.com/gochberg/solute_spectrum.

## APPENDIX A

### Digital phantoms

Water: $R_{1}$ = 1 1/s, $R_{2}$ = 20 1/s.

Solutes: $R_{1}$ = 1 1/s, $R_{2}$ = 20 1/s, f = 0.001.

Diagonal 2.

δω = 1.2, 3.6 ppm.
k = 316, 10 000 1/s.

Square 4.

δω = 1.2, 3.6, 3.6, 1.2 ppm.
k = 316, 316, 10 000, 10 000 1/s.

Square 9.

δω = 1.2, 2.4, 3.6, 3.6, 3.6, 2.4, 1.2, 1.2, 2.4 ppm.
k = 316, 316, 316, 1778, 10 000, 10 000, 10 000, 1778, 1778 1/s.

Square 9 + MT.

Identical to square 9 plus an added pool with ${\delta \omega }_{\mathrm{mt}}$ = −2.35 ppm, $k_{\mathrm{mt}}$ = 49 1/s, $f_{\mathrm{mt}}$ = 0.1, $R_{2\mathrm{mt}}$ = 6000 1/s, and $R_{1\mathrm{mt}}$ = 1 1/s.

δω separation

δω = 2.6, 3.4, 4.0, 4.4 ppm.
k = 1000, 1000, 1000, 1000 1/s

k separation

δω = 3.0, 3.0, 3.0 ppm.
k = 316, 1000, 5623 1/s.

Extreme square 9 + MT.

δω = 1.2, 2.4, 3.6, 3.6, 3.6, 2.4, 1.2, 1.2, 2.4 ppm.
k = 31.6, 31.6, 31.6, 1778, 31 623, 31 623, 31 623, 1778, 1778 1/s.
MT pool with ${\delta \omega }_{\mathrm{mt}}$ = −2.35 ppm, $k_{\mathrm{mt}}$ = 49 1/s, $f_{\mathrm{mt}}$ = 0.1, $R_{2\mathrm{mt}}$ = 6000 1/s, and $R_{1\mathrm{mt}}$ = 1 1/s.

Neuronal solutes (^16^ to nearest 0.1 ppm).

Site = Glc 1, Glc 2, Glc 3, Glc 4, Glc 5, MI, Cr, PCr 1, PCr 2, GABA, Tau, Glu, Gln 1, Gln 2, Gln 3, amides.
δω = 2.2, 2.9, 1.1, 1.4, 0.7, 1.0, 2.0, 1.9, 2.6, 2.9, 3.2, 3.2, 2.2, 2.9, 3.2, 3.5 ppm.
k = 3860, 4750, 3940, 2560, 950, 2090, 810, 67, 126, 6900, 49 600, 7480, 17, 49, 22 880, 221/s.

### Chemical phantoms

PBS buffer at pH = 7.4, room temperature. 60 mM PCr; 20 mM MI; 40 mM GABA; 17.1 mM Cr.

### Acquisition

#### CEST simulations

$B_{0}$ = 15.2 T (unless stated otherwise), SNR = 500 (unless stated otherwise).

$n_{\mathrm{acq}}$ = 150; 30 Δω values (−5, −4.4, −3.8, … −1.4, −0.8, 0.8, 1.0, 1.2, … 5.0 ppm); 5 $\omega_{1}$ values (0.5, 1.0926, 2.3875, 5.2170, 11.4 μT).

#### CEST experiments

Identical to simulations, except that experiments have variations in Δω and $\omega_{1}$ as determined by WASABI fits, calibration of long versus short pulse amplitudes, and coil limitations that are incorporated into the fit (e.g., 0.57, 1.254, 2.736, 5.928, 11.742 μT in one case). Additionally, 20 data points with ineffective irradiation (at 200 ppm) are spread through the acquisition to provide normalizers.

#### $R_{1\rho }$ simulations

$B_{0}$ = 15.2 T, SNR = 500.

$n_{\mathrm{acq}}$ = 600; 60 Δω values (−5, −4.4, −3.8, … −0.8, −0.2, 0.0, 0.1, 0.2, … 5.0 ppm); 10 $\omega_{1}$ values (0.5, 0.7077, 1.0017, 1.4178, 2.0068, 2.8404, 40 203, 5.6904, 8.0542, 11.4 μT).

### Linear fitting

#### CEST

A is a 150 × 1043 matrix.

Bins: $n_{\text{bins}}$ = 1043; 726 (solutes), 315 (MT), 2 (direct).

MT bins: δω = − 5:0.5:5 ppm, k =13, 26, 52 1/s, $R_{2}$ = 500, 1581, 5000, 15 811, 50 000 1/s.

Solute bins: δω = 0.8, 1.0, 1.2, … 5.0 ppm, k = 33 logarithmically spaced between 10 and 10^5^ 1/s.

#### $R_{1\rho }$

A is a 600 × 1967 matrix.

Bins: $n_{\text{bins}}$ = 1967; 1650 (solutes), 315 (MT), 2 (direct).

MT bins: same as in CEST.

Solute bins: δω = 0.1, 0.2, 0.3, … 5.0 ppm, k: same as in CEST.

### L1 fitting parameters (for Lasso fits only)

Bin normalizations: 0.001 (solute bins), 0.1 (MT), 1 ($R_{1}$), 25 ($R_{2},\text{simulations}$), 10 ($R_{2},\text{experiments}$), L1 minimization weighting: λ = 0.005.
